# Knowledge Among Junior Doctors About the Collection and Transport of Samples for Tuberculosis Diagnosis in a Medical College Hospital in Coastal Karnataka, India

**DOI:** 10.7759/cureus.42935

**Published:** 2023-08-04

**Authors:** Jeshela Jamal, Kibballi M Akshaya, Hegde Pavithra

**Affiliations:** 1 Department of Community Medicine, Yenepoya Medical College, Mangalore, IND

**Keywords:** knowledge, resident doctors, diagnosis, samples, tuberculosis

## Abstract

Background

The accuracy of the TB diagnosis depends on the quality of the collected and transported samples. Inadequate knowledge and practices regarding the collection and transport of sputum samples can lead to false-negative results and delay the diagnosis and treatment of TB. This study was conducted to assess the knowledge of residents and interns about the collection and transport of sputum and other samples for the diagnosis of tuberculosis in a medical college hospital.

Methods

This was a cross-sectional study conducted among 120 medical interns and postgraduate residents of a medical college hospital in coastal Karnataka, India. Participants were interviewed using a pre-designed and structured questionnaire. The data was analyzed using IBM Corp. Released 2015. IBM SPSS Statistics for Windows, Version 23.0. Armonk, NY: IBM Corp. Descriptive statistics like the mean, standard deviation, and chi-square test were used. A p-value less than 0.05 was considered statistically significant.

Results

Most of the study participants (76, 63.3%) belonged to the age group of 22 to 25 years and were interns. Nearly three-quarters of the participants (85, 70.8%) were female. Based on the mean score, 69.2% of the participants exhibited good knowledge. Among the socio-demographic characteristics, being a postgraduate resident was associated with good knowledge about the collection and transport of samples (p-value < 0.05).

Conclusion

Seven out of ten participants had good knowledge about the collection and transport of sputum and other samples for the diagnosis of tuberculosis. Strengthening the training of this category of health workers needs to be prioritized.

## Introduction

Tuberculosis (TB) is one of the top 10 causes of death from a single infectious agent (above HIV/AIDS) globally, with an estimated 10.6 million people getting ill with TB and 1.4 million deaths among people with no HIV co-infection in 2021 [1]. Despite immense progress in medical sciences and improved health care facilities, TB remains one of the biggest challenges in developing countries. India alone will contribute over 1.9 million incident TB cases in 2021 [2].

Early and accurate diagnosis is critical for effective TB control, and sputum microscopy remains the primary diagnostic tool in many resource-limited settings. However, the accuracy of the TB diagnosis depends on the quality of the collected and transported samples. Inadequate knowledge and practices regarding the collection and transport of sputum samples can lead to false-negative results and delay the diagnosis and treatment of TB. TB control will be possible only if timely diagnosis and proper drug treatment are made available [3].

Appropriate sample collection and transportation play an important role in the diagnosis of TB. Any error from the beginning of patient preparation, sample collection, and transportation of the sample will lead to an error in diagnosis. The precision of the laboratory results is based on the quality of the samples. Pre-analytical errors are one of the main reasons for the poor quality of samples, which, in turn, is caused by poor knowledge and practice among healthcare workers regarding the same. This applies to sample collection for the diagnosis of TB as well [4].

Improper handling and transport of specimens can cause the death of mycobacteria and lead to false-negative culture results. Unless specimens are collected with the utmost care and promptly transported to the laboratory under the proper conditions, the advantages of culture will not be fully utilized. Safety during the transportation of potentially infected specimens is also critical; incorrect packaging or handling of specimens may be dangerous as it can cause the spread of tuberculosis infection [5]. During specimen collection, it is important to prevent exposure to potential sources of contamination, such as tap water, as the presence of environmental mycobacterium may cause false-positive smear and/or culture results [6].

By providing continual training and educating medical and paramedical staff about the various errors, they can be minimized or eliminated. There is limited literature and studies available on knowledge and practice among junior doctors regarding the collection and transportation of TB samples. This study was done to assess the awareness of residents and interns about the collection and transport of sputum and other samples for the diagnosis of tuberculosis in a medical college hospital in coastal Karnataka, India.

## Materials and methods

Study design: This cross-sectional study was conducted at a tertiary care hospital attached to a medical college in coastal Karnataka, India, between March 2022 and February 2023.

Study setting and population: The study was conducted among the medical interns and postgraduate residents of a medical college hospital located in Mangalore, Karnataka state, India. The hospital has 1248 beds and caters to patients from central and coastal Karnataka and the northern part of Kerala in southern India. There was a total of 413 junior doctors during the study period who fulfilled our inclusion criteria. Junior doctors were either interns or residents from postgraduate specialties except for Anatomy, Physiology, Biochemistry, Pharmacology, Forensic Medicine, and Radiology, where TB-related samples were not handled.

Data collection and analysis: The required sample size of 122 was calculated based on the mean percentage of correct answers about TB diagnosis among medical university students from a previous study (35.7%), a relative error of 25%, and a 10% non-response rate [7]. A total of 120 participants, including 76 interns and 44 residents, participated in this study.

The data was collected using a pre-designed and structured questionnaire. The questionnaire included socio-demographic details, followed by questions related to awareness regarding the collection and transport of pulmonary and extrapulmonary samples for the diagnosis of tuberculosis. The questions were structured based on the guidance documents for sputum collection and transportation from Indian and international contexts [8,9]. Later, the questionnaire was validated for its content by three subject experts. The research questionnaire was distributed as a Google Form link via WhatsApp to the interns and residents. A participant information sheet, which contained all the information about the research, was provided to all participants through the link in the Google Form.

The Google Form responses were downloaded in comma-separated value (CSV) format, and the data captured was cleaned further. IBM Corp. Released 2015. IBM SPSS Statistics for Windows, Version 23.0. Armonk, NY: IBM Corp. was used to process and analyze the data. Statistics like frequencies, proportions, mean, standard deviation, and the chi-square test were used. Participants were categorized into those with good knowledge (a score of more than six) and those with poor knowledge (a score of six or below). A p-value less than 0.05 was considered statistically significant.

Ethics: Ethics clearance was obtained from the institutional ethics committee (Number 888, dated 18th December 2021). Informed consent was obtained from all the participants.

## Results

The majority of the study participants (76, 63.3%) belonged to the age group of 22 to 25 years, followed by 26- to 30-year-olds (30, 25%). Nearly three-quarters of the participants (85, 70.8%) were female. Interns constituted 76 (63.3%) of the study population. Forty-nine (40.8%) participants had undergone training or sensitization on TB diagnosis and management at least once in the last two years. The correct responses by the residents and interns to various questions are mentioned in Table 1.

**Table 1:** Frequency and percentage of correct responses by interns and residents for questions on the collection and transport of samples for the diagnosis of tuberculosis in a medical college hospital in coastal Karnataka, India (N = 120)* *Responses to each question were expressed as 'Yes' or 'No'.

Responses termed correct	Frequency	Percentage
The ideal amount of sputum to be collected is 3 to 5 ml	83	69.2
A satisfactory sputum sample is mucoid or mucopurulent	110	91.7
For a patient with presumptive pulmonary TB, there must be a one-hour gap between the two spot samples	42	35.0
Ideally, sputum specimen collection is to be done in an open space	58	48.3
The appropriate container for sample collection for the CBNAAT test is a falcon tube	32	26.7
Samples should be transported as quickly as possible from the collection site to the laboratory	49	40.8
The ideal temperature for storing the sputum sample in case of a delay in transporting it to the laboratory is 2°C to 6°C	60	50.0
A minimum of 20 to 50 ml of pleural fluid or bronchioalveolar lavage (BAL) must be obtained for the CB-NAAT and microbiological testing	80	66.7
The medium of transport for transbronchial and other biopsies in cases of presumptive TB for culture and CB-NAAT tests is 0.9% sterile normal saline	77	64.2
The medium of transport for biopsy specimens for histopathological examination in cases of presumptive TB is formalin	71	59.2
Gastric lavage is collected in the early morning on an empty stomach, and the specimen is neutralized using 100 mg of sodium bicarbonate and transported	93	77.5
In the case of urine samples, three early morning samples need to be collected	12	10

The minimum, maximum, and mean scores of the participants were 3, 10, and 6.3 (SD=1.588), respectively. Based on the mean scores, 69.2% of participants had good knowledge.

The Chi-square test was used to determine the association between sociodemographic variables and awareness about the collection and transport of sputum and other samples for the diagnosis of tuberculosis. Among the sociodemographic characteristics, being a postgraduate resident was associated with good knowledge about the collection and transport of samples (p-value < 0.05) (Table 2).

**Table 2:** Association of the socio-demographic variables with awareness of interns and residents about the collection and transport of sputum and other samples for the diagnosis of tuberculosis in a medical college hospital in coastal Karnataka, India (N=120)

Socio demographic variables	Groups	Poor knowledge n= 37 (%)	Good knowledge n= 83 (%)	p-value
Gender	Male	8 (22.9)	27 (77.1)	0.225
	Female	29 (34.1)	56 (65.9)	
Category of doctors	Interns	29 (38.2)	47 (61.8)	0.022
	Postgraduate residents	8 (18.2)	36 (81.8)	
Undergone any training/sensitization on TB diagnosis and management in the last two years	Yes	17 (34.7)	32 (65.3)	0.447
	No	20 (28.2)	51 (71.8)	

## Discussion

This is one of the few studies from the Indian context that has looked into knowledge about the collection and transport of sputum and other samples for the diagnosis of tuberculosis among interns and postgraduate residents. This study assessed knowledge regarding both pulmonary and extrapulmonary sample collection and transportation. The limitations of this study include a small sample size from a single site; hence, it has limited generalizability. We did not apply proportional probability sampling for the selection of the study participants.

Almost 7 out of 10 participants (69.2%) had a good knowledge score about the collection and transport of sputum and other samples for the diagnosis of tuberculosis. However, in settings with a high burden of TB, like India, it is expected that all the residents and interns handling potentially infectious samples have good knowledge levels. Nearly three-fourths of the participants had adequate knowledge about the volume of sputum to be collected. The participants lacked knowledge in certain aspects of sample collection, like the duration of the gap between two spot samples (35%) and an appropriate container for the collection of the sample for the cartilage-based nucleic acid amplification test (CB-NAAT) (26.7%). It is also necessary that the residents educate the patients regarding the quantity and quality of sputum so that there can be an accurate diagnosis, especially when microscopy is used. Least-correct responses were observed regarding the collection of samples in the case of presumptive genito-urinary TB (10%). This may be because urine samples are rarely used in TB diagnosis. These are the specific components that need to be included in the training and sensitization programs at the institutional level. Residents were found to have better scores compared to those of the interns, and this association was found to be significant. This could be because residents are more involved in the collection and transportation of various TB samples than interns, providing an edge in working knowledge. Three out of four residents in the United States knew the correct method to obtain a sputum sample when evaluating a patient for pulmonary TB as per the CDC guidelines [10]. A study from north India revealed that doctors in the public sector were better aware of the required number of sputum samples and the sampling method than their counterparts in the private sector [11]. This could be attributed to their training and retraining.

With regards to the extrapulmonary samples, two thirds of the participants were aware of the minimum amount of pleural fluid or bronchioalveolar lavage that needs to be collected for microbiological and CB-NAAT tests. Three out of five participants gave correct answers to the questions related to the media of transport for TB samples. In contrast to this, knowledge (25.4%) and practice (15.9%) about transportation of infectious substances among doctors were low in a study from Khartoum state, Sudan [12]. Another study from north India that focused on specimen collection among the clinicians found deficiencies in the collection and transportation of some extrapulmonary TB samples [13].

Only one in four respondents was aware of the correct type of container to be used for sample collection for the CB-NAAT test in our study. This finding on knowledge regarding CB-NAAT testing was lower than in the study conducted by Yadav et al. among residents, in which 58.3% of residents had satisfactory knowledge regarding CB-NAAT testing [14].

Medical college hospitals are involved in the National TB Elimination Program (NTEP) of India so that presumptive TB cases, including extrapulmonary ones that are difficult to diagnose and treat, are managed with the provision of quality care. Samples from presumptive TB patients are precious, especially when they are extrapulmonary. Keeping these in mind, the residents and interns, who are usually in the fine line of contact with the patients, need to know the basics of sample collection and transport. Strengthening the training of this category of health workers needs to be focused on. One good opportunity for this is to address them at the start of the internship/postgraduate course. Periodic retraining will definitely be an added benefit. Pictorial standard operating procedures and job aids need to be developed by the TB program with a focus on sample collection and transport for dissemination to medical colleges and other tertiary care hospitals.

## Conclusions

Seven out of ten participants had good knowledge about the collection and transport of sputum and other samples for the diagnosis of tuberculosis. However, there is room for improvement. Residents were found to have better scores compared to the interns. There is a need to focus on training and retraining of the residents and interns so that we are able to obtain good samples.
